# Tailoring π--d Magnetic Interactions in Metallated Porphyrin Nanotapes

**DOI:** 10.1002/anie.202515342

**Published:** 2025-11-04

**Authors:** Roberto Robles, Shayan Edalatmanesh, Qiang Sun, Pascal Ruffieux, Roman Fasel, Luis M. Mateo, Giovanni Bottari, Tomás Torres, Nicolás Lorente

**Affiliations:** ^1^ Centro de Física de Materiales CFM/MPC (CSIC‐UPV/EHU) Donostia‐San Sebastián 20018 Spain; ^2^ Nanotech@surfaces Laboratory Empa ‐ Swiss Federal Laboratories for Materials Science and Technology Dübendorf 8600 Switzerland; ^3^ Materials Genome Institute Shanghai University Shanghai 200444 China; ^4^ Departamento de Química Orgánica Universidad Autónoma de Madrid Madrid 28049 Spain; ^5^ IMDEA‐Nanociencia Campus de Cantoblanco Madrid 28049 Spain; ^6^ Institute for Advanced Research in Chemical Sciences (IAdChem) Universidad Autónoma de Madrid Madrid 28049 Spain; ^7^ Donostia International Physics Center (DIPC) Donostia‐San Sebastián 20018 Spain

**Keywords:** π‐d Magnetic interaction, Kondo effect, On‐surface synthesis, Porphyrins, Scanning probe microscopy

## Abstract

Molecular assemblies based on porphyrins (Pors), specifically Por nanotapes (NTs) containing magnetic metal ions, offer a versatile platform to explore magnetic interactions arising from the electronic interplay between π‐conjugated ligands and transition metal *d*‐orbitals. Using on‐surface synthesis under ultra‐high vacuum, we synthesized π‐extended PorNTs of different lengths incorporating magnetic metal ions such as Fe and Co on Au(111). We resolved their atomic structure using scanning tunneling microscopy (STM) and non‐contact atomic force microscopy (nc‐AFM). Differential conductance (dI/dV) measurements, interpreted by extensive density functional theory calculations and theoretical modeling, reveal two distinct magnetic behaviors for the Fe‐ and Co‐based systems. In FePorNTs, the magnetic interactions are dominated by strong Fe–ligand ferromagnetic coupling and weak antiferromagnetic Fe–Fe coupling. By contrast, CoPorNTs exhibit stronger Co–Co antiferromagnetic exchange and weaker Co–ligand coupling, with Kondo screening evident at the ligand sites. Our findings underscore the profound influence of metal centers, ligands, and substrate interactions on the magnetic and electronic properties of PorNTs, establishing these assemblies as interesting building blocks for low‐dimensional magnetism and future spintronic or quantum‐material applications.

## Graphical abstract

To understand how π‐radicals and d‐electrons interact within molecular nanostructures, we have studied metallated porphyrin nanotapes synthesized on gold surfaces. We reveal element‐specific results: with Fe centers the ferromagnetic π‐d exchange dominates, while with Co centers the metal–metal exchange overpowers the π‐d exchange, with Kondo‐screened ligand spins, highlighting porphyrin nanotapes as a versatile platform for designer 1D magnetism.

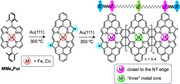

## Introduction

The nanomagnetism arising from the presence of unpaired π‐electrons in organic π‐conjugated systems has garnered significant attention in recent years.^[^ ^1^, ^2^, ^3^, ^4^, ^5^, ^6^, ^7^, ^8^, ^9^, ^10^, ^11^, ^12^, ^13^, ^14^ ^]^ The exceptional properties of carbon atom's π‐electrons, including long spin coherence times and lengths, make carbon‐based systems highly advantageous for spintronic applications.^[^ ^15^, ^16^ ^]^ In particular, nanographenes exhibit large magnetic exchange couplings, ensuring magnetic stability even under practical room‐temperature conditions.^[^ ^3^, ^5^, ^6^, ^8^, ^10^ ^]^

Meanwhile, the spin–orbit coupling and the resulting magnetic anisotropy energy associated with *d*‐ or *f*‐block elements are integral to conventional inorganic magnetic materials, which underpin applications such as permanent magnets and information storage devices. Previous studies investigating coordinating complexes have demonstrated significant magnetic interactions between the π‐electrons of open‐shell organic structures and the *d*‐electrons of transition metals such as Fe and Co, highlighting the fundamental importance of π--d coupling in defining their magnetic properties.^[^ ^17^, ^18^ ^]^ However, a detailed atomic‐scale understanding of π--d magnetic interactions in metal–organic structures remains elusive.

Two main requirements are essential for designing such hybrid magnetic systems. First, the organic structure must exhibit a π‐conjugated magnetic or open‐shell electronic structure. Second, the π‐network must interact with d‐orbitals to facilitate π--d magnetic interactions. Realizing these criteria within a single molecule is challenging due to the inherent instability and poor solubility of organic open‐shell compounds. Porphyrins (Pors) stand out as versatile molecular platforms for this purpose, owing to their planar structure, extended aromatic π‐network, and their ability to chelate transition metals within their core, potentially enabling strong π--d interactions.^[^ ^19^, ^20^, ^21^, ^22^ ^]^

In addition to their intrinsic properties, in multiPor systems, the nature of the Por–Por linkage significantly influences the electronic properties of Por oligomers.^[^ ^23^ ^]^ The degree (singly, doubly, or triply connected),^[^ ^24^ ^]^ site (e.g., β–β, *meso*–β, or *meso–meso*),^[^ ^25^ ^]^ and chemical nature of the linker (e.g., C─C single bond or π‐spacers)^[^ ^26^ ^]^ are key factors that determine the electronic structure and transport properties of these systems. Among these Por‐based ensembles, *meso–meso*, β–β, β–β triple linkages show high degree of π‐conjugation and low HOMO–LUMO gaps, resulting in planar, fused Por nanotapes (NTs) with remarka ble transport properties.^[^ ^27^, ^28^ ^]^ However, their synthesis in solution typically involves multi‐step processes with extremely low yields and stability issues, especially for longer oligomers.^[^ ^28^ ^]^

To overcome these challenges, on‐surface synthesis under ultra‐high vacuum conditions has emerged as a promising strategy.^[^ ^29^, ^30^ ^]^ This approach facilitates the fabrication of planar π‐conjugated systems, which are otherwise unattainable by solution synthesis. Simultaneously, it allows for detailed in situ characterization of structural, electronic, and magnetic properties of these nanostructures via local‐probe techniques such as scanning probe microscopy.^[^ ^31^, ^32^, ^33^, ^34^, ^35^ ^]^

On‐surface synthesis has already been employed to create various one‐dimensional (1D) and two‐dimensional (2D) Por networks, focusing on their structural, electronic, and topological properties.^[^ ^36^, ^37^, ^38^ ^]^ Within this context, the on‐surface synthesis of triply‐fused oligomers is also achievable, as demonstrated by previous reports on porphine dimers,^[^ ^39^ ^]^ Por dimers spaced by short graphene segments,^[^ ^40^, ^41^ ^]^ Zn‐based PorNTs,^[^ ^42^ ^]^ anti‐aromatic square‐type Pors,^[^ ^43^ ^]^ Au‐coordinated Por chains,^[^ ^44^ ^]^ and covalent PorNTs featuring two laterally fused Pors.^[^ ^45^ ^]^

Although metalloPor monomers have already provided atomic‐scale insight into π–d interactions,^[^ ^46^ ^]^ how these interactions evolve in extended NTs with multiple coupled metal centers remains largely unexplored and is the focus of the present study. Here, we extend the investigation of on‐surface synthesized, triply‐fused Por oligomers to Fe‐ and Co‐centered assemblies of up to six metalloPor units. Systematically examining the role of these metal centers enables us to track the evolution of π--d magnetic interactions as 1D molecular structures are built. Our findings provide new insights into the interplay between π‐ and d‐electrons, offering a pathway toward the development of novel topological matter and adding up to the effort of building structures with tailored magnetic properties.^[^ ^47^, ^48^ ^]^ Furthermore, the molecular nature of Por‐based assemblies provides new tools to advance on‐surface synthesis by enabling the creation of previously unattainable structures. These efforts mark an important step toward fabricating 1D molecular systems supported on solid substrates, with profound implications for both fundamental research and applications in quantum materials and spintronics.

## Results and Discussion

**Figure 1:**
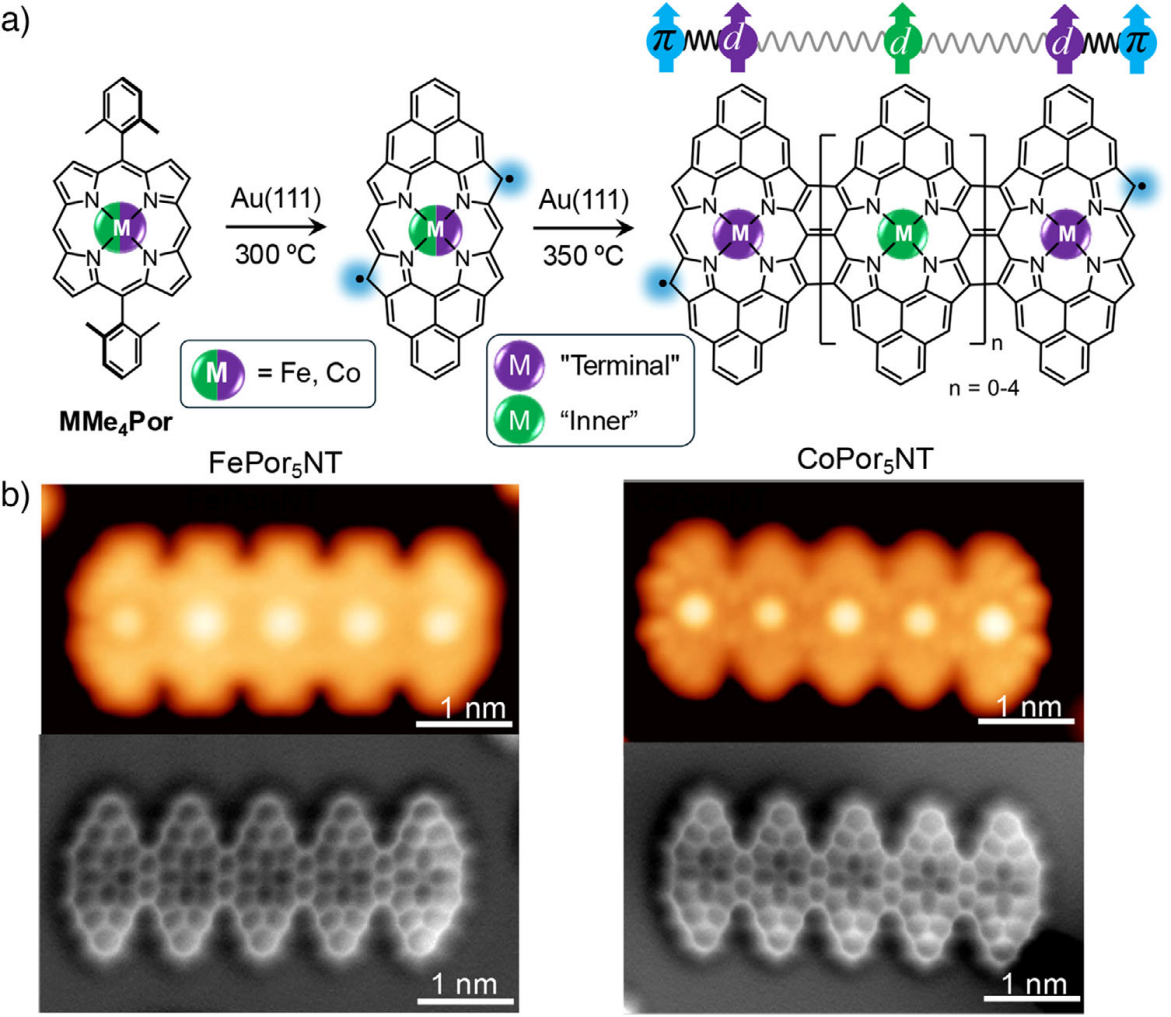
On‐surface synthesis and characterization of PorNTs. a) Scheme depicting the fabrication of the PorNTs and the magnetic interactions taking place within the structures due to the presence of unpaired π and d electronic orbitals in the Por‐based oligomers. b) Constant current STM (top) and nc‐AFM (bottom) images of ${\mathrm{FePor}}_{5}\mathrm{NT}$ (left) and ${\mathrm{CoPor}}_{5}\mathrm{NT}$ (right).

A full description of the experimental procedures and the two theoretical approaches, (i) density functional theory (DFT) and (ii) model Hamiltonians, is given in the Supporting Information (SI). Figure 1 offers a concise overview of the PorNT fabrication workflow (see panel a) and representative images obtained via scanning tunneling microscopy (STM) and non‐contact atomic force microscopy (nc‐AFM) with a CO‐functionalized tip of the on‐surface synthesized ${\mathrm{FePor}}_{5}\mathrm{NT}$ and ${\mathrm{CoPor}}_{5}\mathrm{NT}$ (see panel b) to orient the reader before the detailed discussion that follows.

**Figure 2:**
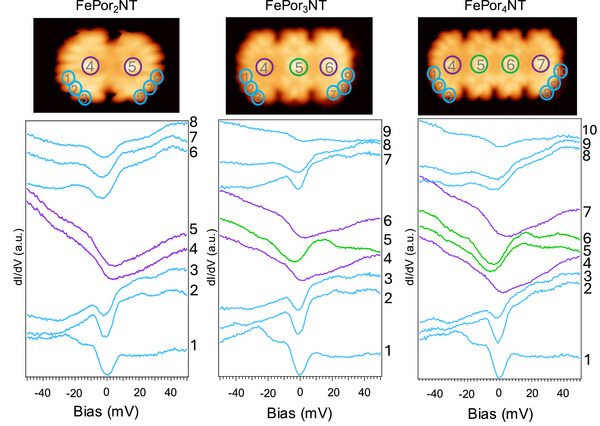
Constant‐current images (top) and differential conductance measurements (bottom) on ${\mathrm{FePor}}_{2}\mathrm{NT}$, ${\mathrm{FePor}}_{3}\mathrm{NT}$, and ${\mathrm{FePor}}_{4}\mathrm{NT}$ on Au(111). The positions at which the STM tip was parked during the differential conductance measurements are marked on the right side of each graph and correspond to the position marked by the same number in the upper STM images. Both inelastic spin excitations and Kondo features are revealed with strong contrast depending on the measurement site.

**Figure 3:**
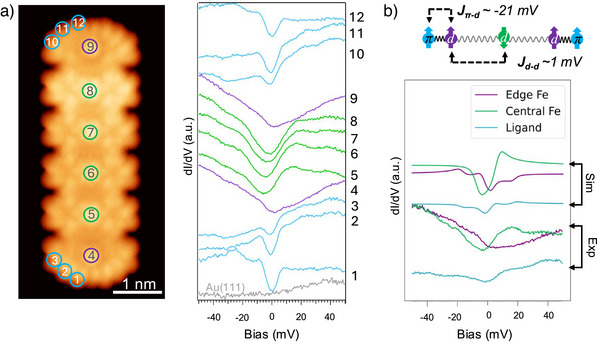
a) Constant current STM image of a ${\mathrm{FePor}}_{6}\mathrm{NT}$ with color‐coded markers. The adjacent curves show representative dI/dV spectra acquired at those positions. b) Schematic illustration of the magnetic interactions considered in ${\mathrm{FePor}}_{3}\mathrm{NT}$ (top): interaction between vicinal Fe ions ($J_{d-d}$) and interaction between the π radical at the PorNT edge and a terminal Fe ion ($J_{\pi -d}$). Simulated (via model Hamiltonians) and experimental dI/dV spectra (5,6,8 in Figure 2) for ${\mathrm{FePor}}_{3}\mathrm{NT}$ are juxtaposed for direct comparison (bottom).

The experimental data for ${\mathrm{FePor}}_{n}\mathrm{NT}s$ (with n=2--6) show three distinct types of dI/dV features depending on where the STM measurement is taking place (see Figures 2 and 3). If the tip is parked on the edge ligand, there is a clear inelastic feature appearing at ±5 mV, and then a faint feature appearing at about 20 mV (see blue traces in Figures 2 and 3). The slope of the dI/dV curve is positive, probably pointing at the presence of the LUMO with a strong weight (highly localized) on the ligands. Moving the tip above the first and last Fe atoms results in a distinct broad gap at positive and finite bias (about 8 mV with an error bar of 5 mV) and an overall negative slope (see purple traces in Figures 2 and 3). Lastly, in the cases of ${\mathrm{FePor}}_{3}\mathrm{NT}$, ${\mathrm{FePor}}_{4}\mathrm{NT}$, and ${\mathrm{FePor}}_{6}\mathrm{NT}$ moving the tip over the inner Fe atoms yields a narrower gap, this time located at negative bias (about ‐4 mV) together with a clear maximum at about 14 mV (see green traces in Figures 2 and 3).

**Figure 4:**
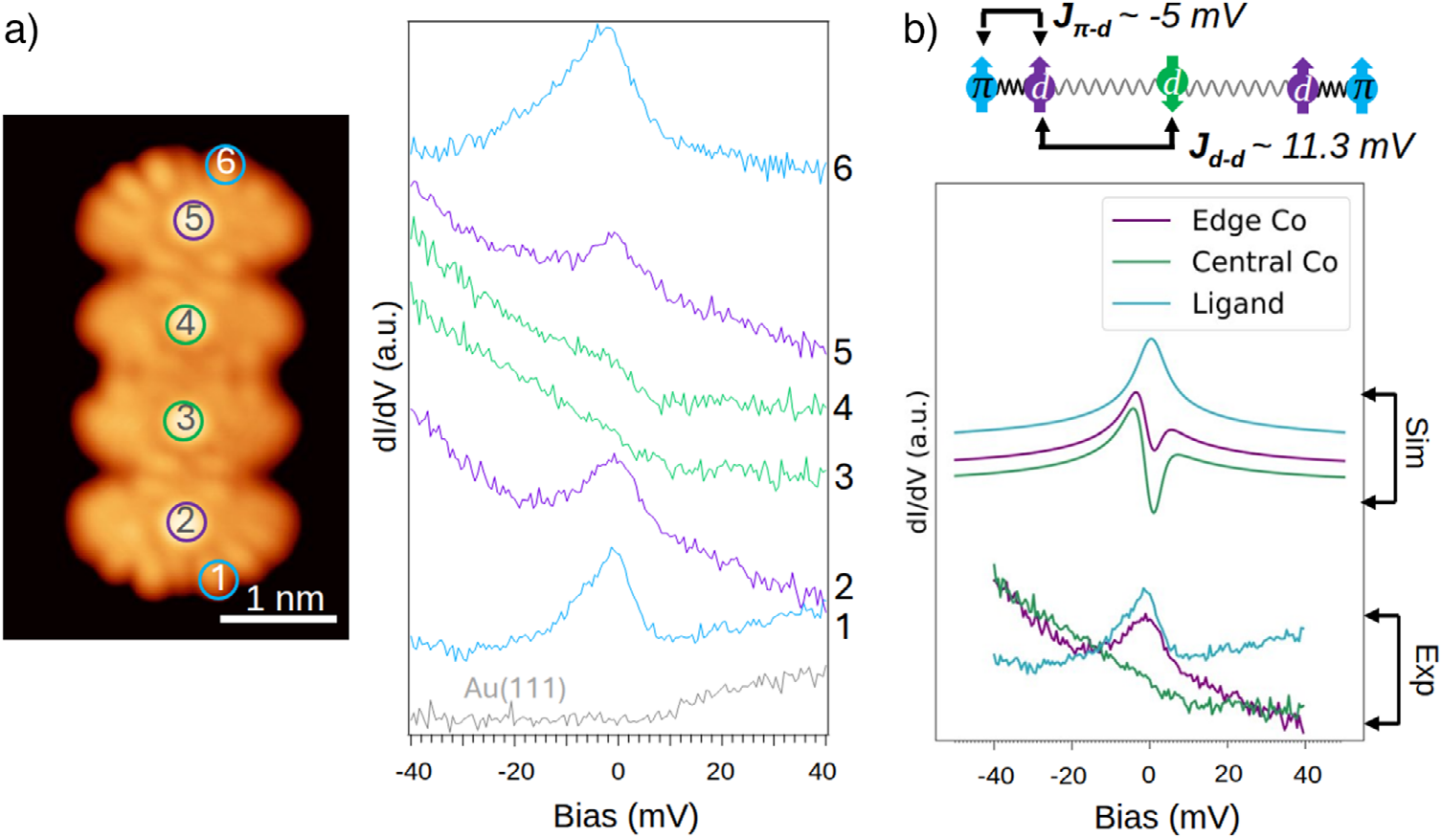
a) Constant current STM image of a ${\mathrm{CoPor}}_{4}\mathrm{NT}$ and differential conductance measurements on the different marked sites of the STM image. b) Schematic representation of magnetic interactions in ${\mathrm{CoPor}}_{3}\mathrm{NT}$ (top) and comparison of model Hamiltonian calculations of the trimer with experimental dI/dV spectra of ${\mathrm{CoPor}}_{4}\mathrm{NT}$ (bottom).

For comparison, we present the results of ${\mathrm{CoPor}}_{4}\mathrm{NT}$ in Figure 4, where we observe substantial differences with respect to the Fe analogue (Figure 3). Although the ${\mathrm{FePor}}_{n}\mathrm{NT}$ spectra exhibit predominantly inelastic features (steps in the differential conductance), the ${\mathrm{CoPor}}_{n}\mathrm{NT}$ spectra (recorded at either the NT edges or on the terminal Co atoms) display pronounced peaks at zero bias, signaling the presence of Kondo screening with the metallic substrate (see blue and purple spectra in Figure 4). The parameters needed to model these distinct spectral behaviors are marked in the top of the b panels in Figures 3 and 4, showing the impact of changing the central transition metal atom on the magnetic interactions.

The magnetic coupling between Fe atoms in ${\mathrm{FePor}}_{n}\mathrm{NTs}$ is observed to be weak. This conclusion arises from both qualitative and quantitative analyses. Qualitatively, in spin chains with significant interatomic magnetic interactions, the energy gap associated with magnetic excitations decreases as the chain length increases.^[^ ^49^ ^]^ However, experimental data shows that the gap at the inner Fe atoms remains constant, regardless of chain size (see green spectra acquired over central Fe atoms in Figures 2 and 3). Quantitatively, simulations reveal that an antiferromagnetic Fe–Fe exchange interaction exceeding 1 meV would produce noticeable changes in the energy gap, which are absent in the experimental spectra. Thus, we infer that the Fe–Fe exchange interaction is smaller than 1 meV (sketch in Figure 3b).

The dI/dV spectra of ${\mathrm{FePor}}_{n}\mathrm{NT}$ reveal additional features near zero bias. These include a gap caused by the inelastic magnetic anisotropy step of Fe atoms (around 5 meV) and a peak most plausibly attributed to Kondo coupling with the substrate. The latter assignment is supported by Frota‐function fits reported in our previous work,^[^ ^42^ ^]^ which establish that such zero‐bias peaks in related metalloPors originate from Kondo screening. Spectral asymmetry arises from electronic scattering involving the highest occupied molecular orbital (HOMO). It should be noted, however, that a rigorous distinction between Kondo resonances and inelastic steps would require systematic temperature‐ or magnetic‐field‐dependent measurements, which lie beyond the scope of the present work. The exchange coupling between an Fe ion and its neighboring diradical π‐extended Por ligand has been estimated to be ∼−21 meV in previous monometallic FePor measurements.^[^ ^46^ ^]^ DFT calculations on ${\mathrm{FePor}}_{2}\mathrm{NT}$ confirm the ferromagnetic character of the Fe–ligand interaction, being significantly stronger than the weak antiferromagnetic Fe–Fe interaction, leading to cooperative alignment of the spins, a behavior commonly referred to as macrospin formation. Model Hamiltonian calculations of the ${\mathrm{FePor}}_{3}\mathrm{NT}$ dI/dV spectra, assuming a −21 meV ferromagnetic Fe–ligand exchange and a 1 meV antiferromagnetic Fe–Fe interaction, are shown in Figure 3b. Although many‐body effects such as substrate hybridization are not included in static calculations, the comparison between these simulations and experimental measurements for ${\mathrm{FePor}}_{3}\mathrm{NT}$ reveals good agreement. The inner Fe atoms show a smaller gap determined by the weak Fe–Fe interaction, while the terminal Fe atoms exhibit a larger, shifted gap due to the stronger Fe–ligand interaction. Magnetic anisotropy may contribute to the sharpening of the features near zero bias by lifting spin‐state degeneracies, although this interpretation remains tentative since the anisotropy values cannot be determined unambiguously from the present data. Moreover, the zero‐bias features assigned to spin excitations (Figures 2 and 3) shift systematically with chain length and are reproduced by our model Hamiltonian simulations. Their broadened appearance prevents the development of sharp valleys at zero bias, which is consistent with the presence of a finite elastic conductance contribution between inelastic steps.

DFT calculations on ${\mathrm{FePor}}_{2}\mathrm{NT}$ structure corroborate these findings, though they highlight challenges in accurately modeling Fe complexes (see SI). The nominal S=1 spin state of Fe atoms in adsorbed systems may differ from gas‐phase configurations due to strong many‐body effects, which complicate computational treatments. These effects likely contribute to the observed variability in computed values for ${\mathrm{FePor}}_{2}\mathrm{NT}s$. Notably, the Fe–ligand exchange interaction remains dominant over Fe–Fe coupling. These two characteristics lead to clear inelastic steps in the dI/dV taken over the ligands, while over the central Fe atoms the features are closer to the combination of local Kondo features and a spin excitation due to the Fe–Fe coupling (see Figure 3b).

We also note that the antiferromagnetic ground states of our finite‐sized Fe‐based PorNTs are in contrast to a previously reported computational study described by DFT and periodic boundary conditions (modeling an infinite system), in which the Fe‐based system was found to favor a ferromagnetic ground state^[^ ^50^ ^]^ by an energy difference $\Delta E_{AFM-FM}=1.56$ eV. To clarify this discrepancy, we have repeated their calculation for the infinite system using our theoretical approach, and instead obtained an antiferromagnetic ground state by $\Delta E_{AFM-FM}=-0.004$ eV. The origin of this large discrepancy is not entirely clear, but we believe that our energy difference in the order of milli‐electronvolts is more reasonable for a magnetic energy difference.

In the CoPorNTs, the magnetic interactions exhibit noticeably different behavior compared to their Fe‐based analogues. Here, the Kondo coupling between Co atoms and the substrate electrons exceeds the exchange interactions between terminal Co ions and the ligand π‐radicals. This dominance of Kondo interactions results in the S=1/2 ligand spins being Kondo screened (see purple and blue spectra in Figures 4 and S5 in the SI).

Experimental dI/dV spectra reveal distinct Kondo signatures at the ligand edges but only faint Kondo peaks at the inner Co atoms, meaning that despite the spin (S=1/2) nature of Co atoms, their Kondo coupling is weaker, particularly for inner Co atoms (see green spectra in Figure 4). This pattern arises because the inner Co atoms couple more strongly to their neighboring Co sites than to the substrate. For comparison, in the previously reported CoPor monomer on Au(111),^[^ ^46^ ^]^ the dominant magnetic fingerprint is a Kondo resonance arising from the singly occupied ${\mathrm{Co}}^{\mathrm{II}}$ $d_{z^{2}}$ orbital, whereas in CoPorNTs the inner Co atoms couple antiferromagnetically, suppressing their Kondo response and giving rise to a competition between Kondo screening and Co–Co exchange. To rationalize these observations, we employed two complementary theoretical approaches. Model Hamiltonian calculations were carried out for ${\mathrm{CoPor}}_{3}\mathrm{NT}$, as depicted in Figure 4b, to capture the essential hierarchy of Co–Co versus Co–ligand interactions in an extended PorNT. These results are compared with the experimental spectra of the tetramer (${\mathrm{CoPor}}_{4}\mathrm{NT}$), as both systems exhibit the same qualitative interaction trends and magnetic hierarchy. In parallel, DFT calculations were performed for ${\mathrm{CoPor}}_{2}\mathrm{NT}$ in both the gas phase and adsorbed on Au(111), as reported in the SI. Although the dimer was chosen for DFT calculations due to the computational cost of treating longer oligomers with sufficient accuracy, our DFT results show reduced coupling values on the surface as compared to the gas‐phase calculations. They reveal a screening effect of the overall magnetic interactions except for the Co–Co interactions (11.29 meV) that seem substantially larger than the Co–ligand ones (−5.04 meV). This behavior matches the above observation that, while the Kondo coupling is larger than the Co–ligand coupling, the trend is reversed for the inner Co atoms. Although the calculated spectra in Figure 4b capture the relative strength of Co–Co versus Co–ligand interactions, they are not expected to reproduce all experimental features quantitatively. Effects such as substrate hybridization, dynamic correlations, and tip‐induced broadening, which strongly influence the measured spectral line features are not fully included in the model Hamiltonian treatment. Therefore, the theoretical spectra should be regarded as illustrating the qualitative exchange trends rather than providing a one‐to‐one match with experiment.

## Conclusion

This study provides a comprehensive analysis of the magnetic interactions and electronic properties of Fe‐ and Co‐based PorNTs synthesized via on‐surface synthesis on Au(111) surfaces. The experimental dI/dV spectra, supported by DFT calculations and model Hamiltonians, reveal significant differences in the magnetic behavior of the two systems, highlighting the crucial role of the transition metal center and substrate effects in determining their properties.

For FePorNTs, the magnetic interactions are governed by strong ferromagnetic coupling between the π radicals at the NT edges and the unpaired d electrons of the terminal Fe atoms, with a coupling strength of approximately −21 meV. The weak antiferromagnetic exchange between vicinal Fe atoms, estimated to be less than 1 meV, results in a system where the Fe–ligand interaction dominates. This leads to macrospin behavior and distinct features in the dI/dV spectra, including magnetic anisotropy steps and suppressed Kondo peaks at ligand sites. Despite computational challenges arising from many‐body effects in Fe complexes, the results demonstrate that ligand exchange is the primary driver of magnetic interactions in these systems.

In contrast, CoPorNTs exhibit markedly different behavior. In this case, the Co–Co exchange interaction is stronger (11.29 meV) and antiferromagnetic, while the Co–ligand coupling is weaker (−5.04meV) and ferromagnetic. Interestingly, the DFT results show that the Au(111) surface profoundly influences the magnetic interactions in CoPorNTs, enhancing the Co–Co exchange relative to Co–ligand coupling and thereby reversing the trend observed in FePorNTs. This reversal of the coupling hierarchy compared to FePorNTs results in Kondo screening of ligand spins and distinct spectral features. The inner Co atoms exhibit weak Kondo signatures due to stronger coupling to adjacent Co atoms than to the substrate. These findings point to the critical influence of substrate effects, which enhance Co–Co exchange interactions relative to Co–ligand coupling.

Overall, our investigations demonstrate substantial differences in the magnetic interactions and electronic structure of FePorNTs and CoPorNTs. Our results underscore the challenges of accurately modeling many‐body interactions in transition‐metal‐based Por assemblies, advance the understanding of π–*d* magnetic interactions, and showcase the potential of these PorNTs for creating novel low‐dimensional magnetic materials. The ability to tailor magnetic properties through choice of metal center, ligand structure, and substrate interactions offers exciting opportunities for the design of novel quantum materials, which could find applications in spintronics and topological matter. Future work could explore extending these systems to larger assemblies and integrating them into device architectures for practical applications where fine‐tuning of magnetic properties is essential.

## Experimental

All experimental details can be found in the Supporting Information.

## Conflict of Interests

The authors declare no conflict of interest.

## Supporting information

Supporting Information

## Data Availability

The data that support the findings of this study are available from the corresponding author upon reasonable request.
