# Supplementary material for: Tailoring π--d Magnetic Interactions in Metallated Porphyrin Nanotapes
Source: Angew Chem Int Ed Engl. 2025 Nov 4;64(52):e15342. doi: 10.1002/anie.202515342 (PMC12723466; doi:10.1002/anie.202515342)
Supplement: Supplementary file 1 — Supporting Information [file ANIE-64-e15342-s001.pdf]

Supporting Information: Tailoring $\pi - d$ Magnetic Interactions in Metallated Porphyrin Nanotapes

Roberto Robles,[a] Shayan Edalatmanesh,*[a] Qiang Sun,[b,c] Pascal Ruffieux,[b] Roman Fasel,*[b] Luis M. Mateo,[d,e] Giovanni Bottari,*[d,e,f] Tomás Torres,*[d,e,f] Nicolás Lorente*[a,g]

[a] Centro de Física de Materiales CFM/MPC (CSIC-UPV/EHU), 20018 Donostia-San Sebastián, Spain

[b] nanotech@surfaces Laboratory, Empa - Swiss Federal Laboratories for Materials Science and Technology, Dübendorf 8600, Switzerland

[c] Materials Genome Institute, Shanghai University, Shanghai 200444, China

[d] Departamento de Química Orgánica, Universidad Autónoma de Madrid, Madrid 28049, Spain

[e] IMDEA-Nanociencia, Campus de Cantoblanco, Madrid 28049, Spain

[f] Institute for Advanced Research in Chemical Sciences (IAdChem), Universidad Autónoma de Madrid, Madrid 28049, Spain

[g] Donostia International Physics Center (DIPC), 20018 Donostia-San Sebastián, Spain
E-mail: shayan.edalatmanesh@ehu.eus,
nicolas.lorente@csic.es,
roman.fasel@empa.ch,
giovanni.bottari@uam.es,
tomas.torres@uam.es

Experimental methods

The experiments were conducted using a commercial low-temperature microscope (Scienta Omicron) for the preparation and *in situ* characterization of the samples using scanning tunneling microscopy (STM) and atomic force microscopy (AFM). All measurements were performed under ultra-high vacuum (UHV) conditions with a base pressure of approximately 2×10^{-10} mbar. Au(111) single crystal substrates were prepared by standard cycles of argon ion sputtering followed by annealing.

Molecular precursors (**CoMe$_4$Por** and **FeMe$_4$Por**) were deposited on a Au(111) substrate via thermal evaporation [1] using a sixfold organic evaporator (Mantis GmbH). STM images of the CoPor and FePor nanotapes (NTs) were acquired at a liquid helium temperature of 4.5 K in constant-current mode unless otherwise specified. Differential conductance ($\mathrm{d}I/\mathrm{d}V$) spectra were measured using the lock-in technique, with $U_{\mathrm{RMS}} = 20$ mV for wide-range spectra and $U_{\mathrm{RMS}} = 0.8$–1 mV for low-bias spectra. Low-bias spectra presented in this study are the average of 8–12 consecutive measurements to ensure accuracy and reproducibility.

Non-contact atomic force microscopy (nc-AFM) images were obtained using a CO-functionalized tip [2] mounted on a quartz tuning fork sensor [3] with a resonance frequency of 23.5 kHz. The peak-to-peak oscillation amplitude was kept below 100 pm for high-resolution imaging. Data analysis and processing were carried out using Wavemetrics Igor Pro and WSxM software.

Spin excitation spectra were analyzed and fitted using specialized software from Ref. [4], while Kondo resonance fitting was performed with a Frota function using Wavemetrics Igor Pro. These methods provided detailed insight into the electronic and magnetic properties of the investigated systems.

Chemicals and solvents were obtained from commercial suppliers (Aldrich, Fluka, Strem, Acros, and Fischer) and used as received, without further purification. All anhydrous solvents were freshly distilled under an argon atmosphere over appropriate drying agents prior to use.

Column chromatography was performed on Merck silica gel 60 (230–400 mesh, 60 Å pore size). Analytical thin-layer chromatography (TLC) was conducted on aluminium sheets precoated with silica gel 60 F254 (Merck), while preparative TLC was carried out using 20 × 20 cm plates (silica gel 60, 0.5 mm thickness, Merck).

^{1}H and ^{1}3C NMR spectra were recorded on Bruker AVANCE 300 MHz, DPX 400 MHz, and DRX 500 MHz spectrometers. Chemical shift values (δ) are reported in parts per million (ppm) relative to tetramethylsilane (TMS) as an internal standard.

UV-visible spectra were acquired using quartz cuvettes with a 1 cm path length in a Varian Cary 50 UV-Vis spectrophotometer. Infrared (IR) spectra were recorded using a Bruker ALPHA Platinum-ATR system.

Mass spectrometry analyses were carried out as follows: MALDI-TOF MS spectra were acquired on a Bruker ULTRAFLEX III MALDI-TOF/TOF instrument; GC-MS spectra were obtained using a Waters GCT mass spectrometer coupled to an Agilent Technologies 6890N gas chromatograph; and APCI-MS spectra were recorded on a Bruker MAXIS II mass spectrometer.

Theoretical Methods

Density Functional Theory Calculations

Density functional theory (DFT) calculations were performed using the VASP code [5]. The projected augmented-wave (PAW) method [6] was used to treat core electrons, while wave functions were expanded using a plane wave basis set with an energy cutoff of 400 eV. The PBE flavor of GGA was used as exchange and correlation functional [7]. Missing van der Waals interactions in this semilocal functional were treated using the Tkatchenko-Scheffler method [8]. The description of Fe and Co d-electrons was improved with the use of the GGA+U method as formulated by Dudarev [9] with effective Hubbard U values $U_{eff} = U - J = 3$ eV for Fe [10], and $U_{eff} = 4$ eV for Co [11]. For the calculation of adsorbed nanotapes the Au(111) surface was simulated using a slab with four Au layers and a vacuum region of 21 Å. The coordinates of all atoms except the two bottom layers were relaxed until forces were lower than 0.02 eV/Å. Charge transfers and magnetic moments were determined by Bader analyses [12].

Using density functional theory (DFT) and model Hamiltonians, we modeled the experimental $\mathrm{d}I/\mathrm{d}V$ spectra observed at low bias ($-50\,\mathrm{mV} < V < 50\,\mathrm{mV}$) in Fe- and Co-based PorNT trimers, which already capture the essential exchange hierarchy and reproduce the experimental trends of longer oligomers. These models build upon our previous work on mononuclear Fe and Co Pors [1] and incorporate several key interactions, as illustrated schematically in Figure 1. The first term accounts for the exchange coupling between the π radicals at the NT edges and the terminal metal atoms (d-shell unpaired electrons). Additional terms include the interatomic exchange interactions between the spins of adjacent metal atoms, the Kondo interaction between local spins and substrate electrons (comprising exchange and Coulomb scattering), and the magnetic anisotropy of the metal spins. The latter is significant for spins larger than $S = 1/2$, and in this study, we consider a longitudinal anisotropy of 5 meV for Fe atoms, consistent with their nominal $S = 1$ spin state [1]. The differential conductance

is approximated as the local density of states in the presence of all possible magnetic interactions that lead to finite-energy excitations in the spectra like atomic spin-flips [4,13,14], zero-energy features like the Kondo effect [15] or very-low-energy excitations like the spinaron [16,17]. In the present approach, we take an effective approach where magnetic interactions are included at the lowest possible perturbation level [4]. In the $\mathrm{d}I/\mathrm{d}V$ spectra calculations, the simulation temperature T was set to $10K$, matching experimental conditions.

Spin-Hamiltonian Calculations

The spin-Hamiltonian calculations were performed using the methodology and code developed by Markus Ternes [4]. It consists in the exact diagonalization of a Hamiltonian written in the product state of N spins that are different because there are transition-metal centers and spins delocalized in the ligands. The different spins were connected via first-neighbor interactions given by a symmetric Heisenberg Hamiltonian,

$$H_J = \sum_i J_i \vec{S}_i \cdot \vec{S}_{i+1}.$$

The spins of the transition metals were subjected to uniaxial anisotropies given by the square of the spins,

$$H_D = \sum_i D_i S_{z,i}^2 + E_i(S_{x,i}^2 + S_{y,i}^2).$$

Finally, a Kondo-like interaction was included between the localized spins and the conduction electrons, and treated to third order in the coupling as explained in Ref. [4]. The role of this term is to create zero-bias anomalies in the IETS, but also to add an overshoot to the inelastic steps due to the opening of a new conduction channel in connection with a spin flip.

The combined effect of the three terms in the spectra of excitations permit us to fit the different interactions and extract valuable information regarding the spin structure of the different porphyrin-based systems studied in this work. We note however, that the Hilbert space dimension of the model scales exponentially as $(2S+1)^n$, which renders simulations of longer oligomers computationally prohibitive. Consequently, we restrict our analysis to short chains, which nonetheless capture the essential interaction hierarchy and allow us to establish meaningful bounds on the metal–metal exchange.

DFT results

$\mathrm{FePor_2NT}$ calculations

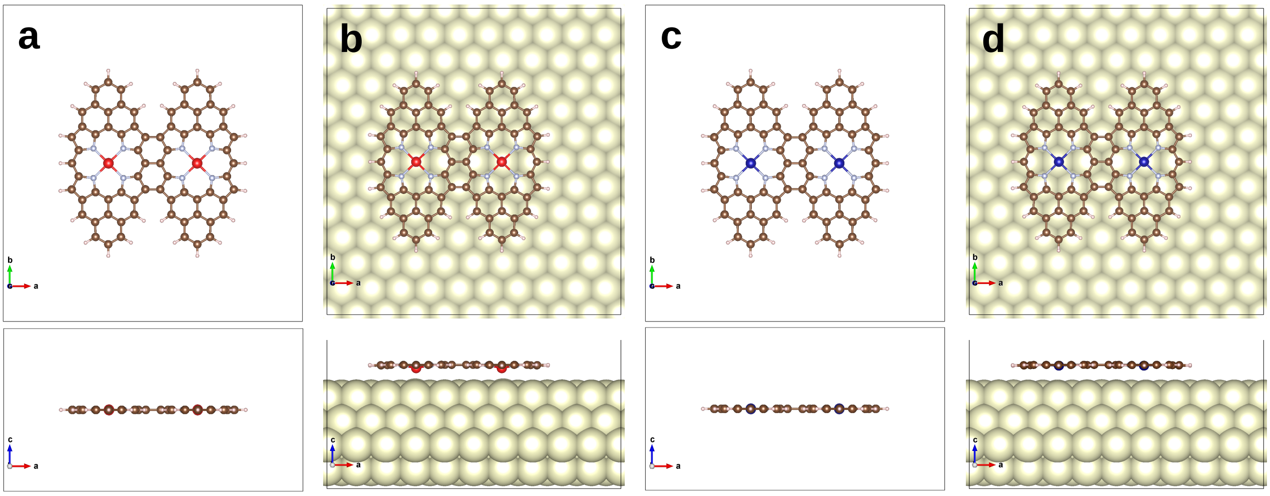

Figure S1. Relaxed structures of **$\mathbf{FePor_2NT}$** (a,b) and **$\mathbf{CoPor_2NT}$** (c,d) in the gas phase (a,c) and on Au(111) (b,d). Red, blue, brown, light blue, pink and yellow spheres represent Fe, Co, C, N, H and Au atoms, respectively. Black lines show the unit cell used in the calculation.

Due to the open-shell structure and many-body character of the FePor_n molecules [18], DFT calculations can only give qualitative results, serving as a guide to the interpretation of the experimental data rather than a quantitative explanation of the findings. Calculations were performed for **$\mathbf{FePor_2NT}$** due to the complexity and

computational cost of describing larger systems. The relaxed structures can be found in Figure S1(a,b). Several spin configurations were converged, permitting us to obtain a qualitative explanation of the interactions at play. The energy differences of different spin configurations can be found in Table S1, while a least-squares fitting of these energies to a first-neighbor Heisenberg Hamiltonian is shown in Table S2. Both in the gas phase and on the Au (111) substrate, the molecules show different spin configurations that change up to about 20 meV among them, the largest energy coming from flipping the spin in the ligands, see Table S1. This behavior matches the finding from the fits to the spectra, where the exchange interaction was larger between Fe and ligands. The Fe-Fe interactions are noticeably smaller. That being said, the meV-scale of the interactions and the reduced accuracy of DFT does not allow us to infer the sign of the interaction with high confidence.

CoPor$_2$NT calculations

In agreement with the case of **FePor$_2$NT**, we obtain a large spin-flip energy difference over the Co-ligand interaction in the gas phase for **CoPor$_2$NT**. However, on the Au (111) substrate we systematically find lower interactions, pointing at a possible screening of the exchange interactions from the surface, see Tables S1 and S2. Given the smallness of the interactions, it is difficult to conclude on the correct tendency of the substrate effect. The DFT interactions then imply a reduced magnetic interaction for CoPor$_n$ that matches the featureless structure of the experimental dI/dV. The relaxed structures are presented in Figure S1(c,d).

Table S1. Energy differences of different spin configurations. Each spin configuration is represented by four letters: first and fourth small-case letters represent the spin state of the ligands, either up (u) or down (d). Second and third upper-case letters represent the spin orientation of the transition metals. The values are given in meV and with respect to the minimum value of each line.

	uUDd	uUDu	uUUu	uDDu	uDUd
**FePor$_2$NT**	0.36	9.30	0.00	22.88	16.95
**FePor$_2$NT**@Au(111)	0.00	8.95	8.03	9.53	19.02
**CoPor$_2$NT**	0.32	9.01	0.00	22.00	16.48
**CoPor$_2$NT**@Au(111)	6.15	3.38	0.51	4.04	0.00

Table S2. Exchange coupling interactions obtained from a least-squares fitting to a first-neighbor's Heisenberg spin Hamiltonian to DFT calculations of the different spin configurations shown in Table S1. Negative couplings refer to ferromagnetic ordering, while positive is antiferromagnetic ordering. Due to the open-shell structure and many-body character of the molecules, these values must be taken as qualitative results.

	$J_{\pi-d}$ (meV)	J_{d-d} (meV)
**FePor$_2$NT**	-9.87	1.29
**FePor$_2$NT**@Au(111)	-2.57	-0.07
**CoPor$_2$NT**	-19.08	4.79
**CoPor$_2$NT**@Au(111)	-1.72	-5.84

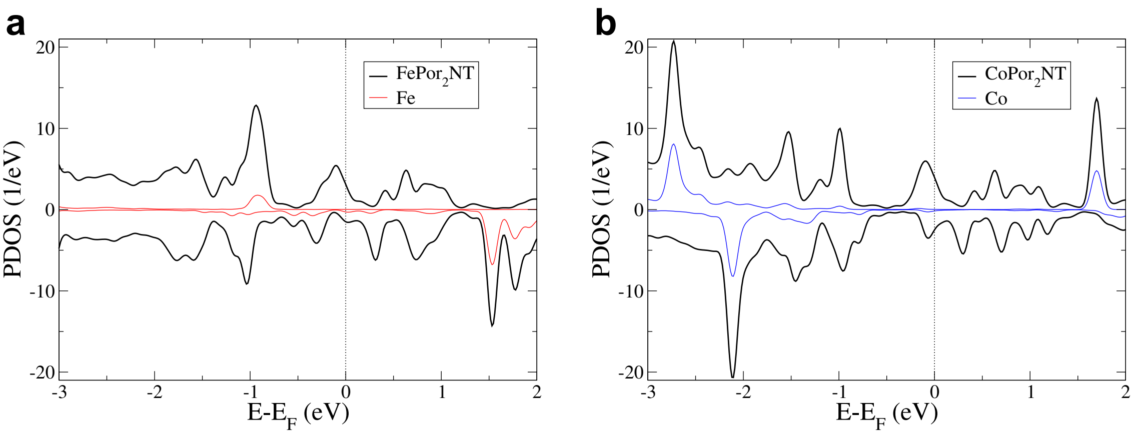

Figure S2. Projected density of states (PDOS) for **FePor$_2$NT** (a) and **CoPor$_2$NT** (b) on Au (111). Black lines represent the states of the nanotape, while red (blue) represent the states of the Fe (Co) center.

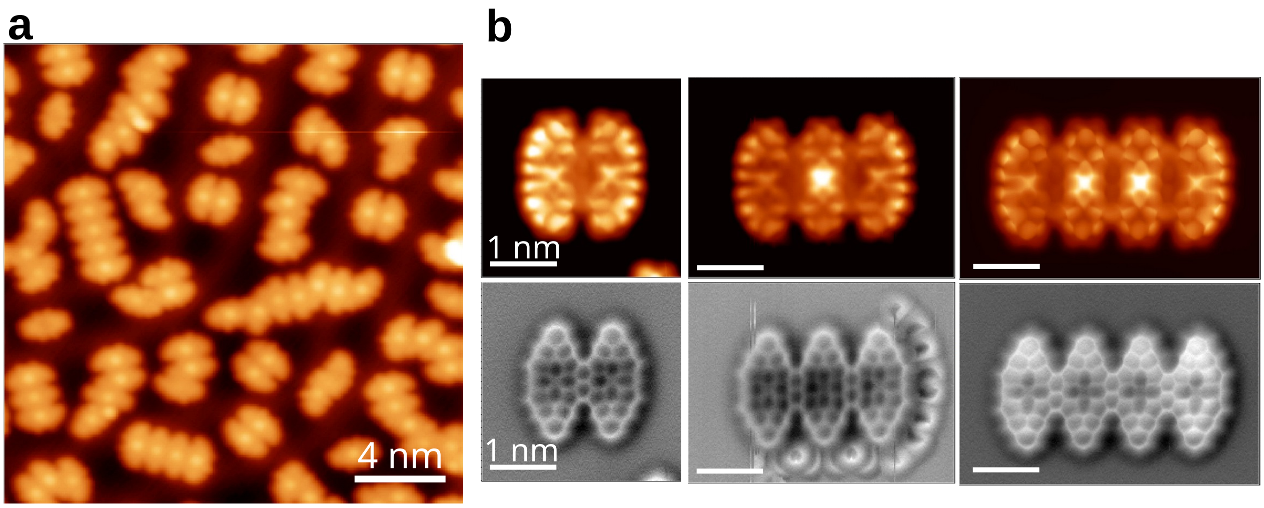

Figure S3. On-surface synthesis of π-extended FePorNTs. (a) Overview STM image of the π-extended FePorNTs grown on Au(111). Scanning condition: Vt $= -1$ V, It $= 20$ pA. (b) Constant-height STM (top) and the simultaneously acquired constant-height nc-AFM (bottom) images of **FePor$_2$NT**, **FePor$_3$NT**, and **FePor$_4$NT** (Vs$= -0.005$ V).

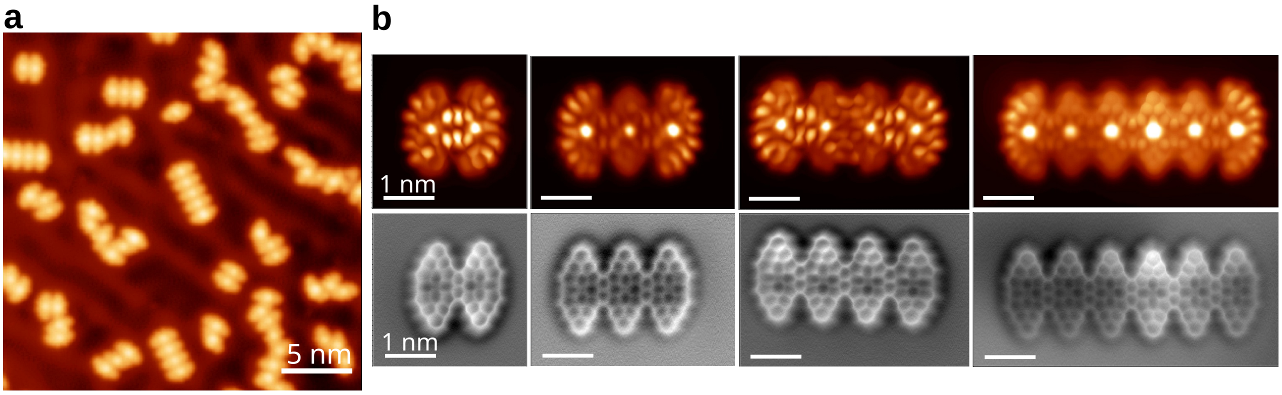

Figure S4. On-surface synthesis of π-extended CoPorNTs. (a) Overview STM image of the π-extended CoPorNTs grown on Au(111). Scanning condition: Vt = -0.2 V, It = 100 pA. (b) Constant-height STM and the simultaneously acquired constant-height nc-AFM images of the **CoPor$_2$NT**, **CoPor$_3$NT**, **CoPor$_4$NT**, and **CoPor$_6$NT** (Vs= -0.005 V).

Figures S3 and S4 show typical AFM and STM images of the different molecular structures formed during the experiments (for FePorNTs and CoPorNTs, respectively). Such data gives an idea of the reproducibility of the molecular structures explored in the present work. The panels (b) of the same figures corroborate their internal architecture and demonstrate that they were fabricated without defects.

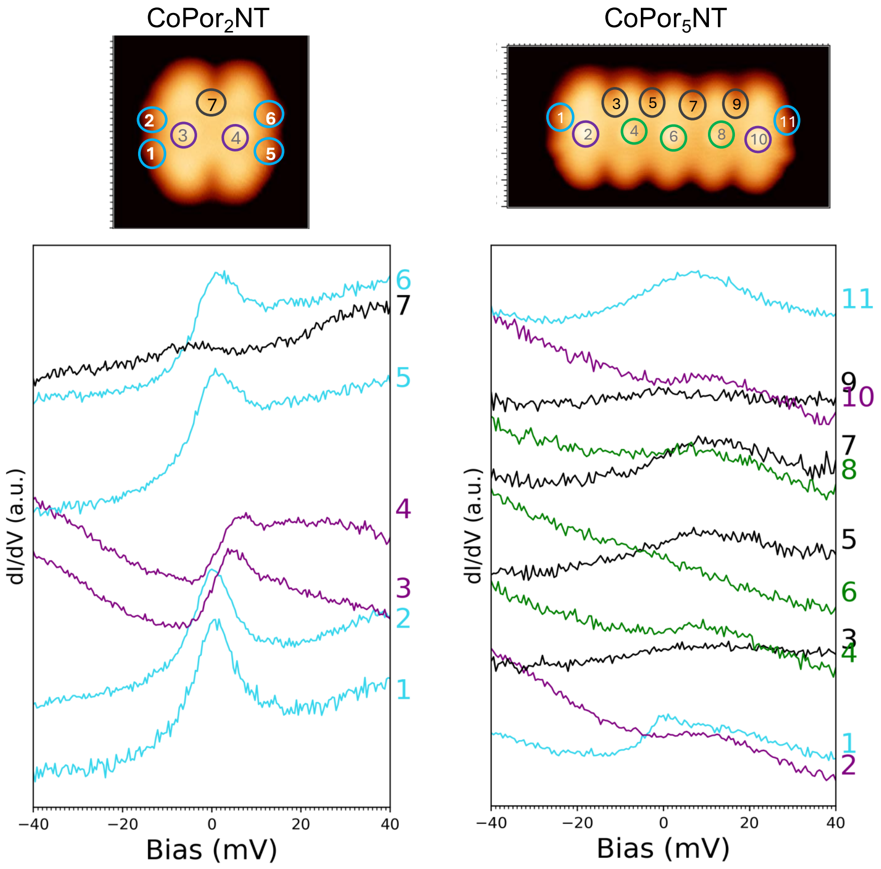

Figure S5. Constant-current images (top) and differential conductance measurements (bottom) on **CoPor$_2$NT** and **CoPor$_5$NT** on Au(111). The positions at which the STM tip was parked during the differential conductance measurements are marked on the right side of each graph and correspond to the position marked by the same number in the upper STM images.

References

[1] Q. Sun, L. M. Mateo, R. Robles, P. Ruffieux, G. Bottari, T. Torres, R. Fasel, N. Lorente, *Adv. Sci.* **2022**, *9*, 2105906.
[2] L. Gross, F. Mohn, N. Moll, P. Liljeroth, G. Meyer, *Science* **2009**, *325*, 1110.
[3] F. J. Giessibl, *Appl. Phys. Lett.* **2000**, *76*, 1470.
[4] M. Ternes, *New J. Phys.* **2015**, *17*, 063016.
[5] G. Kresse, J. Furthmüller, *Comput. Mater. Sci.* **1996**, *6*, 15.
[6] G. Kresse, D. Joubert, *Phys. Rev. B* **1999**, *59*, 1758.
[7] J. P. Perdew, K. Burke, M. Ernzerhof, *Phys. Rev. Lett.* **1996**, *77*, 3865.
[8] A. Tkatchenko, M. Scheffler, *Phys. Rev. Lett.* **2009**, *102*, 073005.
[9] S. L. Dudarev, G. A. Botton, S. Y. Savrasov, C. J. Humphreys, A. P. Sutton, *Phys. Rev. B* **1998**, *57*, 1505.
[10] P. M. Panchmatia, B. Sanyal, P. M. Oppeneer, *Chem. Phys.* **2008**, *343*, 47.
[11] U. G. E. Perera, H. J. Kulik, V. Iancu, L. G. G. V. Dias da Silva, S. E. Ulloa, N. Marzari, S.-W. Hla, *Phys. Rev. Lett.* **2010**, *105*, 106601.
[12] W. Tang, E. Sanville, G. Henkelman, *J. Phys.: Condens. Matter* **2009**, *21*, 084204.
[13] A. J. Heinrich, J. A. Gupta, C. P. Lutz, D. M. Eigler, *Science* **2004**, *306*, 466.
[14] J.-P. Gauyacq, N. Lorente, F. D. Novaes, *Prog. Surf. Sci.* **2012**, *87*, 63.
[15] M. Ternes, A. J. Heinrich, W.-D. Schneider, *J. Phys. Condens. Matter* **2009**, *21*, 053001.
[16] J. Bouaziz, F. S. M. Guimarães, S. Lounis, *Nat. Commun.* **2020**, *11*, 6112.
[17] F. Friedrich, A. Odobesko, J. Bouaziz, S. Lounis, M. Bode, *Nat. Phys.* **2024**, *20*, 28.
[18] A. Calvo-Fernández, M. Kumar, D. Soler-Polo, A. Eiguren, M. Blanco-Rey, P. Jelínek, *Phys. Rev. B* **2024**, *110*, 165113.
